# High-Throughput Gene and Protein Analysis Revealed the Response of Disc Cells to Vitamin D, Depending on the VDR FokI Variants

**DOI:** 10.3390/ijms22179603

**Published:** 2021-09-04

**Authors:** Alessandra Colombini, Paola De Luca, Davide Cangelosi, Carlotta Perucca Orfei, Enrico Ragni, Marco Viganò, Michela Malacarne, Mauro Castagnetta, Marco Brayda-Bruno, Domenico Coviello, Laura de Girolamo

**Affiliations:** 1 Orthopaedic Biotechnology Lab, IRCCS Istituto Ortopedico Galeazzi, 20161 Milan, Italy; deluca.paola@grupposandonato.it (P.D.L.); carlotta.perucca@grupposandonato.it (C.P.O.); enrico.ragni@grupposandonato.it (E.R.); marco.vigano@grupposandonato.it (M.V.); laura.degirolamo@grupposandonato.it (L.d.G.); 2 Laboratorio di Genetica Umana, IRCCS Istituto Giannina Gaslini, 16147 Genoa, Italy; davidecangelosi@gaslini.org (D.C.); michelamalacarne@gaslini.org (M.M.); coviello@unige.it (D.C.); 3 Laboratorio di Istocompatibilità/IBMDR, Ospedali Galliera, 16128 Genoa, Italy; mauro.castagnetta@galliera.it; 4 Scoliosis Unit, Department of Orthopedics and Traumatology-Spine Surgery III, IRCCS Istituto Ortopedico Galeazzi, 20161 Milan, Italy; marco.brayda@spinecaregroup.it

**Keywords:** intervertebral disc, vitamin D receptor gene polymorphism, vitamin D, inflammation, gene profile, protein profile

## Abstract

Vitamin D showed a protective effect on intervertebral disc degeneration (IDD) although conflicting evidence is reported. An explanation could be due to the presence of the FokI functional variant in the vitamin D receptor (*VDR*), observed as associated with spine pathologies. The present study was aimed at investigating—through high-throughput gene and protein analysis—the response of human disc cells to vitamin D, depending on the *VDR* FokI variants. The presence of FokI *VDR* polymorphism was determined in disc cells from patients with discopathy. 1,25(OH)_2_D_3_ was administered to the cells with or without interleukin 1 beta (IL-1β). Microarray, protein arrays, and multiplex protein analysis were performed. In both FokI genotypes (*FF* and *Ff*), vitamin D upregulated metabolic genes of collagen. In *FF* cells, the hormone promoted the matrix proteins synthesis and a downregulation of enzymes involved in matrix catabolism, whereas *Ff* cells behaved oppositely. In *FF* cells, inflammation seems to hamper the synthetic activity mediated by vitamin D. Angiogenic markers were upregulated in *FF* cells, along with hypertrophic markers, some of them upregulated also in *Ff* cells after vitamin D treatment. Higher inflammatory protein modulation after vitamin D treatment was observed in inflammatory condition. These findings would help to clarify the clinical potential of vitamin D supplementation in patients affected by IDD.

## 1. Introduction

Vitamin D supplementation has been proposed in several pathological contexts to improve skeletal and non-skeletal health, but a considerable body of clinical trials has failed to establish clear evidence of benefit [1].

In general, to establish the clinical success of a hormonal supplementation, the mechanism of action of the hormone should be firstly in vitro defined and then the safety and effectiveness of its administration should be validated in vivo.

Considering the intervertebral disc degeneration (IDD)-related pathologies, few in vitro studies reported the presence of human cells expressing the vitamin D receptor (*VDR*) inside the disc, which are able to respond to the hormone by modulating proliferative and metabolic pathways [2,3]. In rat annulus fibrosus (AF) cells vitamin D through VDR ameliorates oxidative stress-induced apoptosis by preserving mitochondrial functions [4]. Furthermore, the same study showed that decreased presence of VDR in discs was associated with age-related IDD in rats [4] and an in vivo study on mice reported a protective effect of the hormone on IDD [5].

The presence of the FokI variant (rs2228570) in the *VDR*, determines the functional modification of the encoded protein, with the shorter polypeptide (*F* allele) couples more efficiently with the transcription factor II B than the longer peptides (*f* allele) and leads to a higher transcriptional rate of vitamin D-dependent genes [6,7]. This could be responsible for differences in the response to the vitamin D supplementation and should be considered in view of a personalized medical treatment. Interestingly, FokI variants in the VDR were observed as associated with spine pathologies [8,9,10,11,12,13], but inconsistent associations have also been reported [14].

In addition to the aforementioned genetic considerations, there are conflicting views concerning the potential of vitamin D in IDD and low back pain treatment. Age-dependent IDD, often associated with osteoporosis—whose treatment aimed at increasing the bone mineral density includes vitamin D supplementation—have been suggested as being responsible of endplate calcification by blocking nutrient and oxygen supply in the disc [15]. Nevertheless, it was observed that subjects with deficiency or insufficiency of vitamin D were more likely to exhibit low back pain than subjects with a normal serum concentration [16,17,18], even if also in this case conflicting evidence is reported [19]. Therefore, the increasingly recognized immunomodulatory role of vitamin D [20,21] could be exploited to target the inflammatory and catabolic process in the IDD [22,23].

In a recent preliminary study on a selected, small panel of markers [24], the regulation of proliferation, metabolism, and inflammatory processes after vitamin D treatment in disc cells was analyzed, with particular attention to the role of FokI *VDR* polymorphism. Vitamin D showed an inhibitory effect on the proliferation and metabolic activity of the disc cells and a pro-apoptotic induction, regardless to the *VDR* genotype, while *Ff* bearing cells showed the most anti-inflammatory and catabolic behavior.

Based on these results, the present study was aimed at investigating through large-scale, high-throughput microarray and protein analysis, the response of disc cells to vitamin D in basal or inflamed condition, depending on the *VDR* FokI variants. The findings of the study could help to clarify the clinical potential of vitamin D supplementation in patients affected by IDD.

## 2. Results

### 2.1. Genes Modulated by Vitamin D Treatment in Homozygous FF and Heterozygous Ff Disc Cells Cultured in Basal Condition

**Figure 1:**
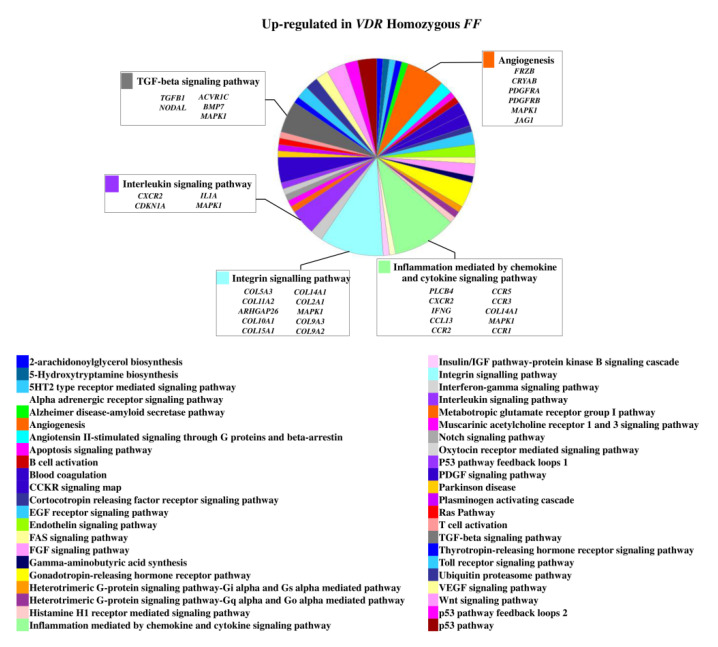
Upregulated genes and related pathways in homozygous *FF* disc cells. Pie chart obtained by Panther.

The 1,25(OH)_2_D_3_ treatment determined an upregulation of 160 genes in homozygous *FF* disc cells and of 113 genes in heterozygous *Ff* cells. In basal condition, 25 genes of those upregulated in *FF* cells were also upregulated in *Ff*. The most relevant upregulated genes in the context of intervertebral disc (IVD) pathophysiology in homozygous *FF* disc cells were *ADAMTS15*, *ACAN*, *BGN*, *KRT18*, *KRT19*, *SOX9*, *IGFBP2*, *IGFBP5*, *IL7* and *TIMP3*, *COL11A2* along with *COL10A1*, *COMP*, *CCR3*, *CCR5*, and *VEGFD* (Figure 1 and Supplementary Table S1), that were also upregulated in *Ff* disc cells.

**Figure 2:**
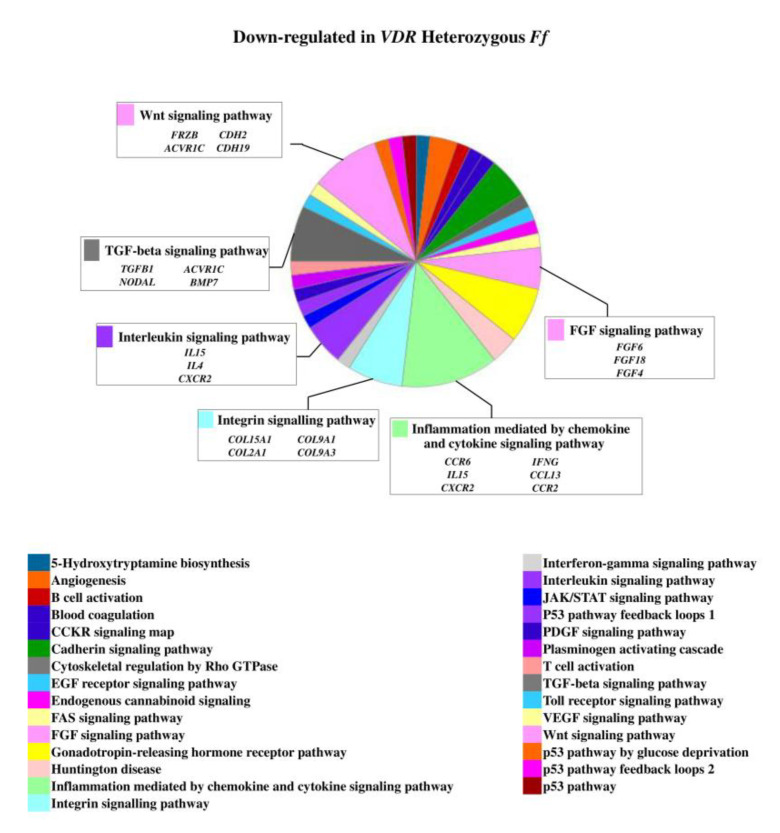
Downregulated genes and related pathways in heterozygous *Ff* disc cells. Pie chart obtained by Panther.

**Figure 3:**
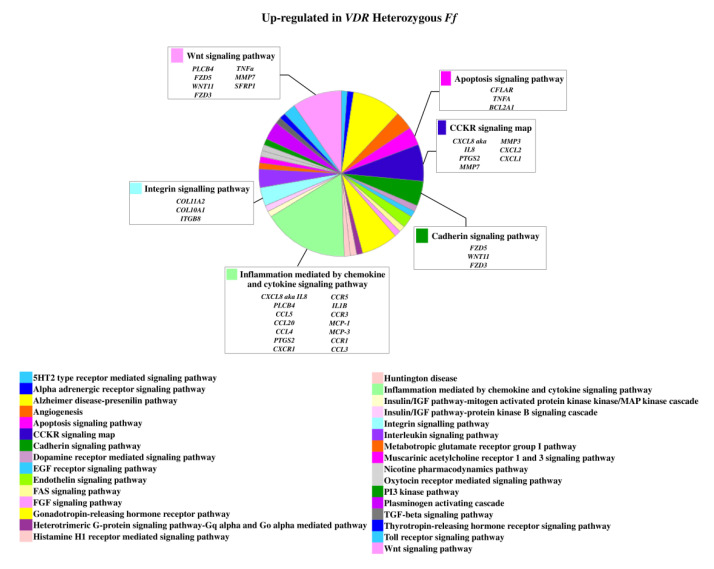
Upregulated genes and related pathways in heterozygous *Ff* disc cells. Pie chart obtained by Panther.

**Figure 4:**
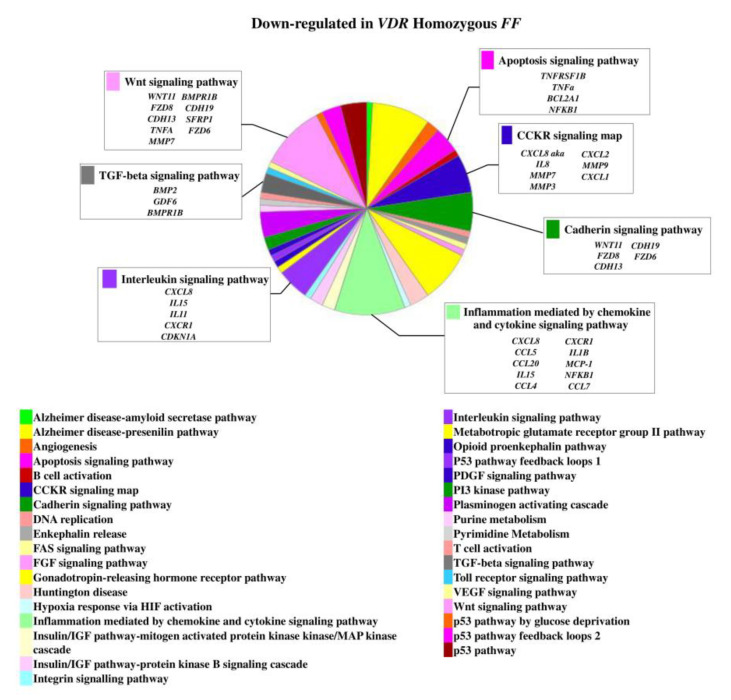
Downregulated genes and related pathways in homozygous *FF* disc cells. Pie chart obtained by Panther.

On the contrary, 49 genes upregulated in *FF* disc cells were downregulated in *Ff* cells, among which *ACVRIC*, *CCL13*, *CCR2*, *COL15A1*, *COL2A1*, *COL9A3*, *CXCR2*, *BMP7*, *DCN*, *FGF18*, *FRZB*, *INFG*, *IL10*, *IL17A*, *IL1R2*, *IL2*, *IL31RA*, *IL5*, *IHH*, *IDO2*, *NODAL*, *TGFB1*, and *TNMD*, together with other 46 genes that were exclusively downregulated in *Ff* such as *KRT14*, *TERT*, and *VEGFR* (Figure 2 and Supplementary Table S1). Among the upregulated genes in *Ff* cells, the most relevant in the context of IVD pathophysiology were *IL1B*, *TNFA*, *MMP3*, *WNT11*, CCL, and CXCL chemokines (Figure 3). Among this gene groups, 65 were instead downregulated in homozygous *FF* cells, including *ADAMTS9*, *BMP2*, many CCL and CXCL chemokines, *FGF7*, *IGF1*, *IL11*, *IL1B*, *IL1RN*, *IL32*, many MMPs, *NOS2*, *TNFA*, *TNFAIP6*, *TNFAIP8*, and *WNT11*, together with other 71 genes exclusively downregulated in *FF* cells, such as *ADAMTS4*, *CSF2*, *HIF1A*, *IDO1*, *IGF2*, *IGFBP1*, *IGFBP3*, *IL15*, and *MMP9* (Figure 4 and Supplementary Table S1).

### 2.2. Genes Modulated by Vitamin D Treatment in Homozygous FF and Heterozygous Ff Disc Cells Cultured in Inflamed Condition

In inflamed condition, *Ff* cells showed to be more responsive in comparison with the ones bearing *FF* genotype. In fact, despite in each group of inflamed homozygous or heterozygous cells the number of up- or downregulated genes was similar, one-third of genes were modulated in *FF* cells in comparison with *Ff* cells.

Under inflammation, the most relevant genes upregulated were *ADAMTS15*, *CCR2*, *CCR5*, *COL15A1*, *COL5A3*, *COL9A1*, *FRZB*, *IDO1*, *IHH*, *IL5*, *IL7*, *KRT19*, *TERT*, *TGFB1*, and *VEGFD* in *FF* and *BMP2*, CCL, and CXCL chemokines and receptors, *CDH19*, *COL11A1*, *COL4A1*, *CSF1*, *CSF2*, *GDF6*, *IGF2*, *IGFBP1*, *IL10*, *IL11*, *IL17A*, *IL1R2*, *IL1RN*, MMPs, *NODAL*, *NOS2*, *TNFA*, *TNFAIP6*, *TNFAIP8*, and *WNT11* in *Ff* (Supplementary Table S1). Conversely, the most relevant downregulated genes were *ADAMTS9*, *CCL13*, *CCR3*, *COL11A2*, *COL2A1*, *COMP*, *FGF18*, *IGF1*, *IL13*, *IL1A*, *IL32*, *KRT14*, *NODAL*, *TNFA*, *WNT11* and *ACVR1C*, *ADAMTS15*, *ADAMTS4*, *BGN*, *CCR1*, *CCR2*, *CCR3*, *COL11A2*, *COL14A1*, *COL2A1*, *COL9A2*, *COL9A3*, *DKK1*, *FGF18*, *FRZB*, *IDO1*, *IGF1R*, *IGFBP2*, *IGFBP5*, *IHH*, *IL13*, *IL1A*, *IL1B*, *IL2*, *TGFB1*, *TGFB3*, *TIMP3*, *VEGFD* in *FF* and *Ff*, respectively.

**Figure 5:**
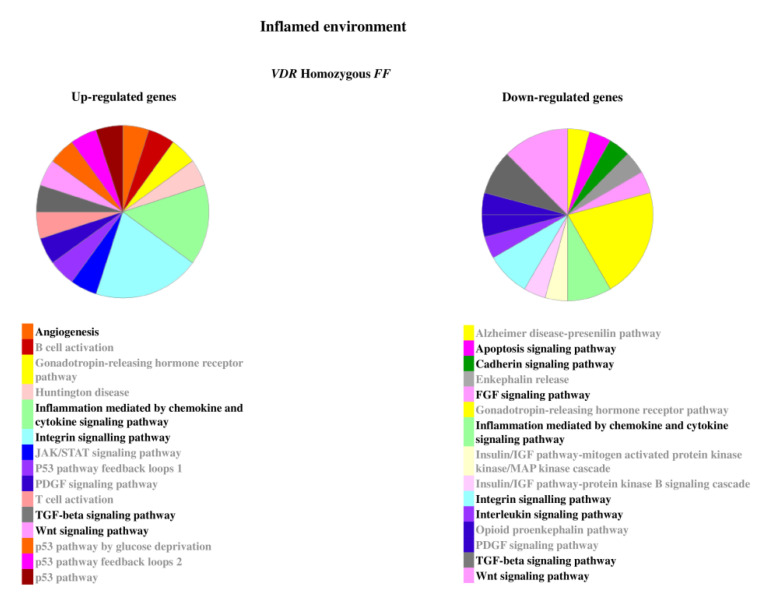
Pathways associated to the genes modulated by inflammatory stimulus in homozygous *FF* disc cells. Relevant pathways are highlighted in black bold. Pie chart obtained by Panther.

**Figure 6:**
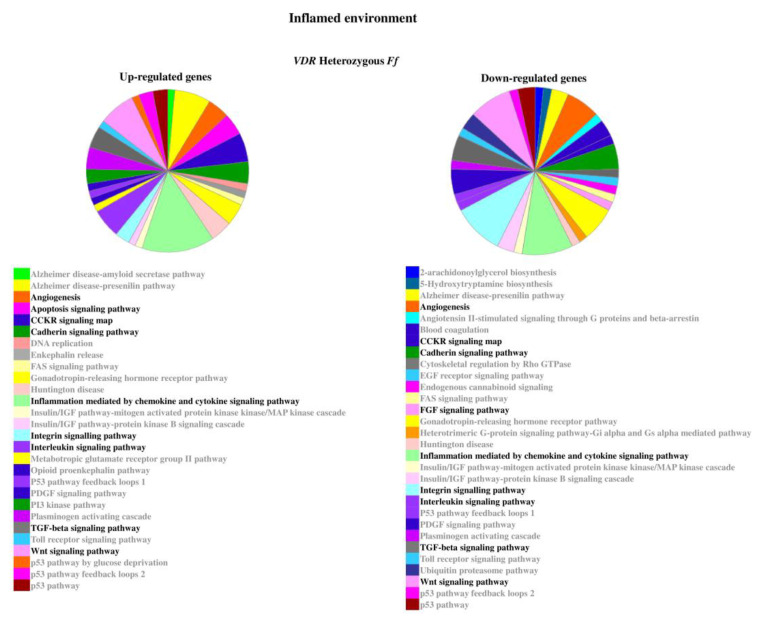
Pathways associated to the genes modulated by inflammatory stimulus in heterozygous *Ff* disc cells. Relevant pathways are highlighted in black bold. Pie chart obtained by Panther.

The relevant pathways associated to the genes modulated by inflammatory stimulus are reported in Figure 5 and Figure 6.

### 2.3. Relevant Pathways Affected by Vitamin D Depending on VDR FokI Genotypes

**Figure 7:**
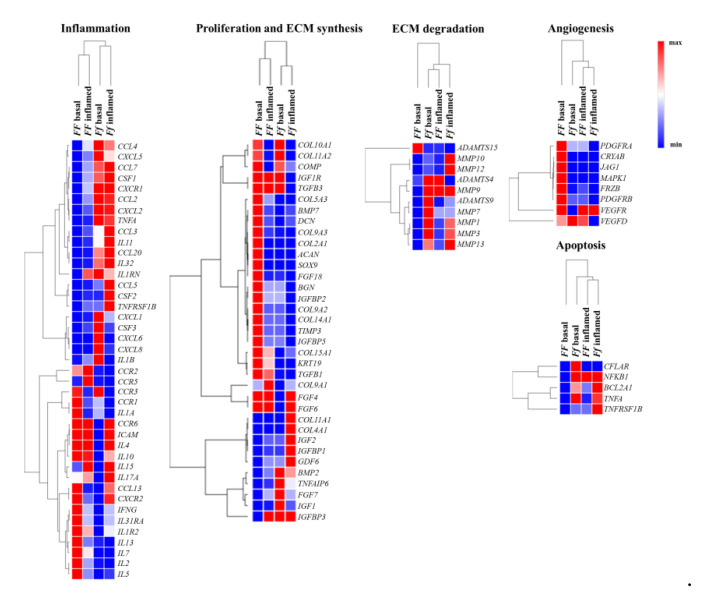
Heat maps shows relevant group of genes differently modulated by vitamin D depending on FokI genotype in basal and inflamed conditions. Heat maps obtained by Morpheus, https://software.broadinstitute.org/morpheus (Date of access 6 April 2021).

Heat maps shows relevant group of genes differently modulated by vitamin D depending on FokI genotype in basal and inflamed conditions (Figure 7).

Vitamin D modulated some genes involved in extracellular matrix (ECM) remodeling in *FF* cells, whereas *Ff* ones appeared less responsive to vitamin D in this regard, although in this cell group two relevant genes having a protective role in disc ECM degradation, *IGF1* and *TNFAIP6*, were highly upregulated instead downregulated in *FF* cells. In both FokI genotypes, vitamin D upregulated genes involved in type II collagen synthesis, and in collagen metabolism in general.

In *FF* cells, inflammation seems to hamper the synthetic activity mediated by vitamin D, whereas it does not affect much the trend of *Ff* cells observed in basal condition. In fact, in *Ff* cells, both in basal and inflamed conditions, there is a general upregulation of genes encoding for enzymes involved in ECM remodeling/catabolism. Conversely, these genes were downregulated in basal and remained substantially unchanged in inflamed condition in vitamin D-treated *FF* cells.

Angiogenic markers appeared upregulated by vitamin D treatment in *FF* cells and oppositely downregulated, with a few exceptions, in *Ff* cells in basal condition, whereas, in both inflamed *FF* and *Ff* cells almost all the angiogenic markers were downregulated.

Interestingly, some of the above-mentioned markers are also involved in the cross-talk between TGFβ/BMP and WNT signaling, playing important roles in the formation of bone and cartilage, and in chondrocytes hypertrophy and appeared modulated by vitamin D in disc cells. 

After vitamin D treatment, a clear downregulation in *FF* and an upregulation in *Ff* cells of both pro- and anti-apoptotic mediators as well as of chemokines and their receptors was observed in basal condition, with non-relevant modifications upon inflammation.

Finally, considering the modulation of inflammation by vitamin D, there is a general up- or downregulation of inflammatory markers in the two groups of *FF* and *Ff* cells which did not allow to identify a clear trend. As a general consideration, IL-1β stimulus seems to switch off the vitamin D activity on all the analyzed genes, particularly in *FF* cells.

### 2.4. Higher Inflammatory Protein Modulation after Vitamin D Treatment Was Observed in Inflammatory Condition and Particularly in Ff Cells

Regardless of the treatment, I-309 (CCL1), IL-1α, IL-4, IL-5, IL-7, IL-10, IL12-p40 (IL12B), IL-13, IL-15, MCP-2 (CCL8), MCP-3, M-CSF, MDC (CCL22), MIG (CXCL9), MIP-1-δ (CCL15), RANTES, SCF (KITLG), SDF-1 (CXCL12), TARC (CCL17), TNFβ, EGF, IGF-1, angiogenin (ANG), and oncostatin M (OSM) were undetected in all the samples. Among these proteins, IL12-p40, MCP-2, MDC, MIG, MIP-1, SCF, SDF-1, TARC, TNFβ, EGF, angiogenin, and oncostatin M showed no changes of the correspondent gene expression.

There was a substantial agreement between gene and protein patterns, as confirmed especially by the high correspondence between undetected/unmodulated genes and proteins. Table 1 shows genes expression and correspondent proteins secretion in disc cells depending on the presence of FokI receptor variants after vitamin D treatment in basal and inflamed condition.

In basal condition, in *FF* cells the vitamin D treatment determined an activation of VEGF and upregulation of PDGF-BB secretion, whereas in *Ff* cells an activation of IL-2 (downregulation of the gene), IL-3, and IL-8 (upregulation of *CXCL8*) secretion. Moreover, in *Ff* cells upregulation of MCP-1 (*CCL2*) secretion and gene expression and inhibition of TGFB1 and IFNγ secretion, along with a downregulation of the correspondent genes, and of leptin were observed.

Upon inflammation in presence of vitamin D, *FF* cells secreted higher amount of TNFα, although the related gene expression appeared downregulated, as well as IL-3. Conversely, PDGF-BB was downregulated and GM-CSF (*CSF2*), IL-1β, TPO, and VEGF secretion were inhibited.

In the same conditions, *Ff* cells showed an activation of the PDGF-BB and leptin, with an inhibition of G-CSF (*CSF3*), IL-2 (confirming the gene downregulation), and IL-3 secretion. Moreover, the secretion of IL-6, IL-8, GRO/GROα (*CXCL1*), ENA-78 (*CXCL5*), GM-CSF, and MCP-1 was downregulated too, albeit for all these markers an opposite upregulation of the correspondent genes was observed.

### 2.5. Validation of the Response to Vitamin D Observed in the Nucleus Pulposus (NP), Annulus Fibrosus (AF), and Cartilaginous Endplate (EP) Highest Responsive Ff Cells

Among the 24 evaluated proteins, IL-17A was the only one undetected in all the samples, both in basal and inflamed conditions. 

**Figure 8:**
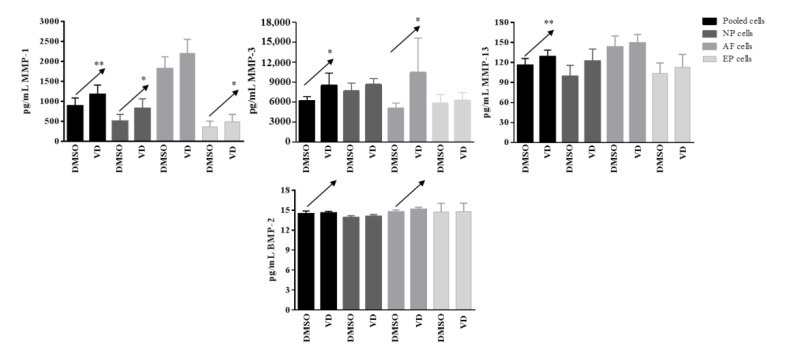
Levels of matrix-metalloproteases and BMP-2 secreted by pooled, NP, AF, and EP cells of 7 donors in basal condition. Arrows indicate significant differences and tendencies in protein secretion of vitamin D treated cells in comparison with untreated cells. * *p* ≤ 0.05, ** *p* ≤ 0.01.

In basal conditions, IL-1β, IL-4, IL-10, IL-12p70, IL-33, IL-1Ra, TNFα, IFNγ, CD40L, and BMP-7 were undetected. Moreover, no changes in IL-2, IL-5, IL-6, IL-13, IL-15, IL-17E, GM-CSF, and CCL20 were observed after vitamin D treatment. Conversely, a significant increased release of MMP-1 after vitamin D treatment was observed in pooled cells (*p* = 0.004), NP (*p*= 0.03), and EP (*p* = 0.03) cells; this increase was also observed for MMP-3 in pooled cells (*p* = 0.03) and AF (*p* = 0.03) cells, whereas for MMP-13 in pooled cells (*p* = 0.005) only. A clear increase, although not significant, was also observed in term of BMP-2 realease in pooled (*p* = 0.08) and in AF (*p* = 0.09) cells (Figure 8).

IL-3 showed no changes both in basal and inflamed conditions after vitamin D treatment.

In inflamed conditions after vitamin treatment, no change in the secretion of MMP-1, MMP-3, BMP-2, and BMP-7 were observed in none of the samples. The secretion of IL-1β, IL-5, TNFα, and CCL20 was observed at single cell population level, whereas in pooled cells a significant decrease was observed for TNFα (*p* = 0.008) and CCL20 (*p* = 0.05). Although not statistically significant, IL-1β, IL-5, and MMP-13 also showed a clear tendency to decrease (*p* = 0.06, 0.07, and 0.07, respectively).

A significant general decrease in the secretion of several other cytokines and inflammatory factors was observed after vitamin D treatment in inflamed condition. Interestingly, this decrease was seen in the pooled cells as well as confirmed in all or some of the single cell populations. GM-CSF appeared decreased in presence of vitamin D in pooled cells (*p* = 0.0003), as well as in all the three single cell populations (NP, *p* = 0.004; AF, *p* = 0.009; EP cells, *p* = 0.03). Similarly, IL-12p70 was decreased in pooled cells (*p* = 0.001), as well as in NP (*p* = 0.005) and AF cells (*p* = 0.03), whereas IL-4 was decreased in pooled cells (*p* < 0.0001) and in NP (*p* = 0.007) and EP (*p* = 0.03). IL-33, IFNγ, CD40L, and IL-13 showed a decrease in pooled cells (*p* = 0.004, *p* = 0.007, *p* = 0.004, and *p* = 0.003, respectively), and in AF cells only (*p* = 0.04, *p* = 0.05, *p* = 0.05, and *p* = 0.03, respectively), whereas IL-10 was decreased in pooled cells (*p* = 0.006) and in NP cells only (*p* = 0.009). Finally, IL-17E showed a decrease mediated by vitamin D in pooled cells (*p* = 0.009) as well as in EP cells only (0.04).

**Figure 9:**
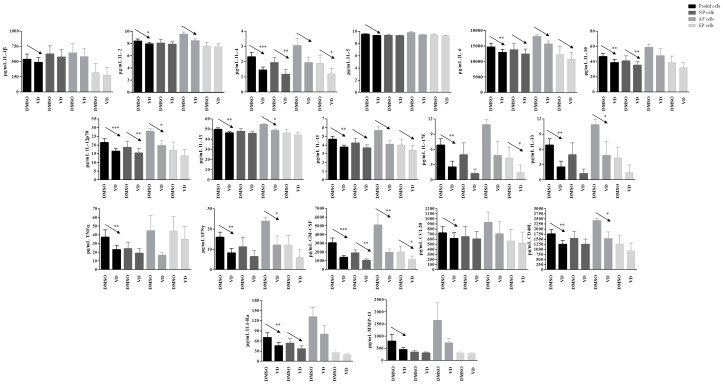
Levels of specific proteins secreted by pooled, NP, AF, and EP cells of seven donors in inflamed condition. Arrows indicate significant differences and tendencies in protein secretion of vitamin D treated cells in comparison with untreated cells. * *p* ≤ 0.05, ** *p* ≤ 0.01, *** *p* ≤ 0.001.

Other molecules/factors such as IL-1Ra, IL-2, IL-6, and IL-15 showed a similar behavior, with statistically significant decrease in the pooled cells (*p* = 0.01, 0.05, 0.002, 0.004, resepctively). The decrease was also confirmed at single cell population level, although not statistically significant, but showing a clear trend (Figure 9).

## 3. Discussion

The present study represents a proof of concept in which the use of omics approaches gave an overall view of the molecular processes putatively affected by vitamin D in the intervertebral disc and endplate. It would be useful to preliminarily identify relevant pathways modulated by vitamin D in this context that should be further analyzed with more focused studies. Overall, it was observed a modulation of common pathways, particularly involved in matrix remodeling and chondrocytes hypertrophy and often regulated in an opposite way depending on the FokI *VDR* genotype. In presence of an inflammatory stimulus, vitamin D action on disc cells appeared less evident, in particular in *FF* cells, confirming that *Ff* cells are more responsive to the hormone, as previously observed [24].

Vitamin D promoted the synthesis of matrix proteins in *FF* cells, also showing a substantial downregulation of enzymes involved in ECM catabolism, except for *ADAMTS15*, a catabolic enzyme that was highly upregulated and generally increased in human degenerated IVD tissue [25,26]. *Ff* cells behaved oppositely, showing a less synthetic and a more catabolic attitude in presence of vitamin D. However, it is worth noting that tumor necrosis factor inducible gene 6 protein (*TNFAIP6*)—having a protective role in cartilage ECM degradation and inflammation [27,28]—was highly upregulated by vitamin D both in basal and inflamed condition only in *Ff* cells.

Among the upregulated markers in *FF* cells there were also those related to angiogenesis, along with hypertrophic markers such as type X collagen, alkaline phosphatase and BMPs, the latter three upregulated in *Ff* cells after vitamin D treatment too. This suggests an involvement of vitamin D in the control of some pathways leading to hypertrophy in disc cells especially bearing the *FF* genotype. Moreover, since IVD is avascular, vessels located near the disc are fundamental for its nutrition, but the presence of neo-vascularization inside the disc is considered associated to the development of pain and degenerative processes. Therefore, it could be interesting to better understand the role of vitamin D in mediating angiogenesis-related processes in the disc through functional studies.

Interestingly, some of the markers modulated by vitamin D treatment in disc cells are also involved in the TGFβ/BMP and WNT signaling pathways which jointly regulate each other and mediate the formation of bone and cartilage by progenitor cells, particularly by skeletal progenitor cells, and are also involved in chondrocytes hypertrophy [29,30,31].

Inflammation is another peculiar aspect to consider during data interpretation. Although the expression of many inflammatory genes was modulated by vitamin D, the number of proteins detectable in the cell culture supernatant was low, suggesting that a low number of inflammatory mediators have a relevant role in this context. Nevertheless, these proteins allowed to explain better the gene modulations and to identify putative feedback mechanisms activated by disc cells. After vitamin D treatment, the pro-inflammatory IL-2 was more secreted by *Ff* cells in basal condition, but inflammation determined a downregulation of both gene expression and protein secretion. A very similar behavior was observed for IL-3, with an increased secretion in *Ff* cells in basal condition and in inflamed *FF* cells, but it is downregulated in inflamed *Ff* cells. Interestingly, the secretion of IL-6, a cytokine known to be highly upregulated by IL-1β treatment [24,32], was downregulated in *Ff* cells after vitamin D treatment, confirming previously published data [24]. A comparable downregulation of the secretion was observed for other chemokines, suggesting again the activation of feedback mechanisms in inflamed *Ff* cells. In basal condition, vitamin D promoted the upregulation of CXCL8 (very highly upregulated also at gene expression level) as well as the secretion of CCL2 in *Ff* cells. On the contrary, both these chemokines were downregulated in inflamed conditions, likewise CXCL1, CXCL5, CSF2, and CSF3. CCL20, a chemokine involved in Th17 response [33], produced by degenerated and IL-17A or TNF-α stimulated NP cells [34], was also upregulated by vitamin D in *Ff* cells, but a concomitant downregulation of its receptor *CCR6* and of the pro-inflammatory *IL17A* was observed. Since the expression of CCR6 and IL-17A was also increased in PBMCs derived from IDD patients and in pathologic IVD tissues [34,35], their downregulation likely represents an attempt of *Ff* cells to limit self-detrimental effects of CCL20-mediated signaling. In addition, IL-17A was undetectable in the supernatant of all *Ff* samples, suggesting that it was not released in a biologically relevant amount.

A general anti-inflammatory attitude of *Ff* cells after vitamin D treatment was suggested also by the downregulation of IFNγ at both gene and protein level and by the upregulation of *IL1RN*, in agreement with the increase of IL-1Ra secretion previously observed in basal condition [24]. On the contrary, the upregulation of *IL1RN* in inflamed cells did not correspond to an increase of IL-1Ra secretion after vitamin D treatment.

As a general conclusion concerning inflammatory markers, *Ff* cells appeared more responsive in the control of inflammation than *FF* cells. In fact, inflamed *FF* cells downregulated the secretion of IL-1β and CSF2, but increased that of TNFα.

Considering TNFα pathway, our data were different in comparison with those reported by an in vivo and in vitro study on mice and their NP cells and showing that vitamin D acts through the inhibition of NF-κB pathway to retard IVD degeneration, modulating inflammatory responses, oxidative stress, apoptosis, and senescence [5]. The inconsistencies between animal and human data might be due to the different cell reaction to the hormone treatment related to the *VDR* variants and in the specie/specificity of the responses. Moreover, the cross-talk with many other pathways and inflammatory cells in vivo, the different cell models (NP for mice vs pooled NP, AF, and EP cells for humans), and inflammatory stimulus (10 fold higher in the mice study) might have led to these differences too.

The main limitation of this study is that no cells bearing the *ff* genotype were collected, due to the low frequencies of this genotype in the Italian population (about 13%), as already published [13]. Moreover, although the NP, AF, and EP cells were isolated and treated separately, then they were pooled and this did not allow discriminating the different responses of the three cell types. However, the aim of this study was to provide an overview of the responses upon stimulation with vitamin D of the disc and endplate as a unique unit, mimicking what would happen in a clinical setting. In the attempt to overcome at least in part this last limitation, a validation of the data observed on the most responsive pooled *Ff* cells was performed in NP, AF, and EP cells obtained from seven donors.

This validation allowed to better explain the results obtained with omics on pooled cells. In general, the trend obtained by pooled cells of seven donors were confirmed in the single NP, AF, and EP cell populations, in particular AF cells showed to be the most responsive. Briefly, the results confirmed previously published [24] and omics data of the present manuscript, where *Ff* bearing cells treated with vitamin D demonstrated a general catabolic behavior, with increase of MMPs release, despite an increase of the anabolic BMP2 in basal condition. An anti-inflammatory attitude of these cells after vitamin D treatment was also confirmed in inflamed environment, with a general decrease of cytokines and inflammatory factors.

## 4. Materials and Methods

### 4.1. Tissue Sample Collection

The study was approved by the Ethic Committee of the San Raffaele Hospital (Protocol GenVDisc Version 1, 20 November 2015) and specimens were collected after obtaining the patient’s informed consent. NP, AF, and EP were collected from L5-S1 discs, Pfirrmann grade IV, of four patients (all women, age range 27–50 y/o, mean age 41 y/o) with diagnosis of discopathy and who underwent discectomy. Six cell populations, three for each donor, were used for the experiments for each genotype.

### 4.2. Isolation and Expansion of Disc and Endplate Cells

To isolate cells, NP, AF, and EP were minced in small pieces and digested under constant agitation with type II collagenase (37 °C, 22 h) (Worthington Biochemical Co, Lakewood, NJ, USA), at the concentration of 224 U/mL for NP, 560 U/mL for AF [36] and 336 U/mL for EP cells [37]. After digestion, the samples were filtered by cell strainers (40 μM for NP and AF, 100 μM for EP) and centrifuged at 1000× *g* for 5 min. The cells were plated at 10^4^ cells/cm^2^ and cultured up to 80–90% of confluence in low glucose (1 mg/mL) Dulbecco’s modified Eagle medium (LG-DMEM, Thermo Fisher Scientific, Waltham, MA USA) supplemented with 10% foetal bovine serum (FBS, Lonza, Basel, Switzerland), 0.29 mg/mL L-glutamine, 100 U/mL penicillin, 100 µg/mL streptomycin, 10 mM Hepes, 1 mM sodium pyruvate (all reagents from Thermo Fisher Scientific, Waltham, Massachusetts, USA) and maintained at 37 °C with 5% CO_2_. Medium was replaced twice a week. When confluent, the cells were detached with 0.05% trypsin/0.053 mM EDTA (Thermo Fisher Scientific, Waltham, Massachusetts, USA) and plated at 5 × 10^3^ cells/cm^2^ until they reached passage 3.

### 4.3. Determination of FokI VDR Genotypes

FokI VDR genotypes of disc cells were determined after genomic DNA extraction with Pure LinkTM Genomic DNA Mini kit (Invitrogen, Carlsbad, CA, USA) and spectrophotometric quantification (NanoDrop, Thermo Fisher Scientific, Waltham, Massachusetts, USA) by using TaqMan SNP Genotyping Assay (Thermo Fisher Scientific, Waltham, Massachusetts, USA) for rs2228570 polymorphism and StepOne Plus instrument (Thermo Fisher Scientific, Waltham, Massachusetts, USA) as previously reported [24]. Half of the patients carried the *FF* while the remaining half carried the *Ff* genotypes.

### 4.4. 1,25(OH)_2_D_3_ and IL-1β Treatment Protocols

NP, AF, and EP cells at passage 3 were detached and seeded at 10^4^ cells/cm^2^ in standard culture medium for 24 h, thereafter the standard medium was replaced with low serum (5% FBS) medium in order to limit the binding between vitamin D and serum protein. After 24 h from the medium shift, 10^−8^ M 1,25(OH)_2_D_3_ or vehicle (0.1% DMSO) (Sigma-Aldrich, St. Louis, MO, USA) with or without 1 ng/mL of IL-1β were added to the cultures. The cells were collected after 48 h of treatment, counted, and pooled per treatment and genotype (2 × 10^5^ cells for each cell type for each genotype), resulting in eight final samples that were maintained at −80 °C until RNA extraction.

### 4.5. RNA Extraction and Quality Assessment

RNA was extracted from lysates of pooled cells by RNeasy Plus Mini Kit (Qiagen, Duesseldorf, Germany). For residual genomic DNA digestion, RNase-Free DNase Set (Qiagen) was used and the isolated RNA was quantified spectrophotometrically (Nanodrop, Thermo Scientific, Rockford, IL, USA). To evaluate the RNA integrity number (RIN), Agilent RNA ScreenTape System (Agilent Technologies, Santa Clara, CA, United States) was used, values ranged from 10 (intact) to 1 (totally degraded) [38]. All RNA samples were intact and showed a RIN value ranging from 9.6 to 10.

### 4.6. Gene Expression Microarray

A custom gene expression microarray was designed through a suitable algorithm of the Agilent Technologies (https://earray.chem.agilent.com/earray/, date of access 12 February 2018) to analyze a huge panel of genes of interest (maximum 3000 genes, including at least five replicates for each gene) [32,39].

Each RNA sample was added with a spike mix (prepared with the One Color RNA Spike-In kit) in order to assess the correct annealing of 10 optimized positive control transcripts to the complementary probes of the array and to evaluate the auto- and cross-hybridization. 

100 ng of RNA was labeled and amplified by Low Input Quick Amp Labeling Kit one-color to obtain cRNA that was purified using RNeasy Plus Mini Kit (Qiagen, Hilden, Germany). The samples were then hybridized on the microarray slide with the Gene Expression Hybridization Kit. The slide was than washed and scanned using SureScan Microarray Scanner to obtain a high-resolution image of the fluorescence values for each probe.

Unless otherwise specified, all the reagents, instruments and software were purchased by Agilent Technologies (Santa Clara, CA, USA).

Data were extracted by Feature Extraction v.12.0 software (Agilent). To evaluate the reproducibility and reliability of microarray experiments, quality control report was generated for each sample. The data analysis was performed by Genespring GX software 14.9 and fold change Fc > 2 or <0.5 in the cells exposed to different treatments were described and considered of interest. Pathway analysis was carried out by Panther database [40] (PANTHER16.0, http://www.pantherdb.org/, 12 February 2018).

### 4.7. Protein Array

The levels of inflammatory mediators in culture media obtained from disc cells, stimulated or not with IL-1β and vitamin D, were evaluated with ELISA-based protein arrays AAH-CYT-3 (RayBio^®^ C-Series, RayBiotech, Peachtree Corners, GA, USA), detection sensitivity up to pg/mL of protein, according to manufacturer’s instruction.

The conditioned media were obtained for NP, AF, and EP cells, from four donors (half cells bearing *FF* and half cells bearing *Ff* genotypes) and pooled according to genotype and treatment.

The membranes were analyzed using ChemiDoc Touch Imaging System (Biorad, Hercules, CA, USA). The results were generated by quantifying the mean spot pixel density using Image Lab Software 2.4 (Biorad). The signal intensities were normalized to the background and positive control and signal intensity results was obtained by the average pixel density of two spots per each inflammatory mediator. The relative concentration of the antigen in the treated sample was proportional to the signal intensity for each control spot. Fold change Fc > 2 or <0.5 in the cells exposed to different treatments were described and considered of interest.

### 4.8. Validation of the Release of Selected Proteins by Luminex^®^ Assays

A panel-relevant proteins identified by gene and protein arrays of pooled samples has been assayed and validated in each single population of NP, AF, and EP cells of seven donors bearing the most responsive *Ff* genotype. 

A custom panel (Human Magnetic Luminex Screening Assay, Bio-Techne, Minneapolis, MN, USA) for the multiple detection of BMP-2, BMP-7, IL-1Ra, IL-2, IL-3, IL-6, IL-17A, MMP-1, MMP-3, and MMP-13 was created. Detection range and sensitivity for these analytes are reported in Supplementary Table S2. A further fixed panel of 17 analytes (Human Discovery Th9/Th17/Th22 Fixed Panel, Bio-Techne) comprising IL-1β, IL-2, IL-4, IL-5, IL-6, IL-10, IL-12 p70, IL-13, IL-15, IL-17A, IL-17E, IL-33, MIP-3α (CCL20), CD40 Ligand, GM-CSF, IFNγ, TNFα was performed to validate some previously analyzed parameters and to add other 14 proteins to the validation, using a more sensitive methods in comparison with a protein array. The range of detection of each protein in the fixed panel is in the order of pg/mL and is reported on the product datasheet.

The assay was performed on a MagPix™ Luminex System (Bio-Rad Laboratories, Inc., Hercules, CA, USA) following the manufacturer instructions.

### 4.9. Statistical Analysis

Data are showed as mean with standard error mean (SEM). Kolmogorov–Sminorv test was perfomed to assess the normal distribution of the data. Student’s *t*-test was performed in case of gaussian distribution whereas Wilcoxon test was performed if data were not normally distributed to establish significative differences between cells treated with vitamin D in comparison with controls. A *p* value ≤ 0.05 was considered significative, 0.09 ≤ *p* > 0.05 was considered as a tendency.

## 5. Conclusions

The strength of the present study was to expand the knowledge of the pathways modulated by vitamin D in IVD cells in relation to the presence of functional variants in VDR by using omics methods. This allowed to overcome the restricted evaluation of panels of small number of molecules, by providing a more complete identification of specific areas of the hormone’s action. These findings pose robust basis for a clinical investigation aimed to assess the potential of the administration of vitamin D in patients affected by disc degeneration. In fact, only this approach would permit to evaluate both the response to the treatment according to the VDR genotype of the patients and the effect of the hormone on matrix remodeling and on hypertrophy/calcification of the tissues involved.

## Figures and Tables

**Table 1:** Protein array and expression of their encoding genes in disc cells depending on the presence of FokI receptor variants after vitamin D treatment in basal and inflamed condition.

		Basal				Inflamed			
		*FF*		*Ff*		*FF*		*Ff*	
Gene	Protein	Gene	Protein	Gene	Protein	Gene	Protein	Gene	Protein
*CXCL5*	ENA-78	−	n.d.	=	n.d.	+	=	+	−
*CXCL1*	GRO/GROα	−	n.d.	+	n.d.	=	=	+	−
*CXCL8*	IL-8	−	n.d.	+	+	=	=	+	−
*CCL2*	MCP-1	−	=	+	+	=	=	+	−
*CCL5*	RANTES	−	n.d.	+	n.d.	=	n.d.	+	n.d.
*CCL7*	MCP-3	−	n.d.	+	n.d.	=	n.d.	+	n.d.
*IL1A*	IL-1α	+	n.d.	=	n.d.	−	n.d.	−	n.d.
*IL1B*	IL-1β	−	n.d.	+	n.d.	=	−	−	n.d.
*IL2*	IL-2	+	=	−	+	=	=	−	−
*IL3*	IL-3	=	n.d.	=	+	=	+	=	−
*IL4*	IL-4	=	n.d.	−	n.d.	=	n.d.	=	n.d.
*IL5*	IL-5	+	n.d.	−	n.d.	+	n.d.	=	n.d.
*IL6*	IL-6	=	n.d.	=	n.d.	=	=	=	−
*IL7*	IL-7	+	n.d.	=	n.d.	+	n.d.	=	n.d.
*IL10*	IL-10	+	n.d.	−	n.d.	+	n.d.	+	n.d.
*IL13*	IL-13	=	n.d.	−	n.d.	−	n.d.	−	n.d.
*IL15*	IL-15	−	n.d.	−	n.d.	=	n.d.	=	n.d.
*IFNG*	IFNγ	+	n.d.	−	−	=	n.d.	=	n.d.
*TNFA*	TNFα	−	n.d.	+	n.d.	−	+	+	n.d.
*CSF1*	M-CSF	−	n.d.	+	n.d.	=	n.d.	+	n.d.
*CSF2*	GM-CSF	−	n.d.	=	n.d.	=	−	+	−
*CSF3*	G-CSF	−	n.d.	+	n.d.	=	n.d.	=	−
*LEP*	Leptin	=	=	=	−	=	=	=	+
*IGF1*	IGF-1	−	n.d.	+	n.d.	−	n.d.	=	n.d.
*PDGFB*	PDGF-BB	=	+	=	n.d.	=	−	=	+
*TGFB1*	TGFβ1	+	n.d.	−	−	+	n.d.	−	n.d.
*VEGF*	VEGF	=	+	=	n.d.	=	−	=	n.d.

Increase (+), decrease (−), unmodulated/undetected genes and unmodulated proteins (=), not detected proteins (n.d.).

## Data Availability

https://osf.io/qbnp8/?view_only=d78deb790b334530a31a71aebf2af8a3, date of access 18 February 2021.

## Supplementary Materials

The following are available online at https://www.mdpi.com/article/10.3390/ijms22179603/s1, Table S1: fold changes (Fc) of genes modulation vitamin D mediated depending on the presence of FokI receptor variants; Table S2: detection range and sensitivity of analytes of the custom Human Magnetic Luminex Screening Assay.
