# Supplementary material for: High-Throughput Gene and Protein Analysis Revealed the Response of Disc Cells to Vitamin D, Depending on the VDR FokI Variants
Source: Int J Mol Sci. 2021 Sep 4;22(17):9603. doi: 10.3390/ijms22179603 (PMC8431769; doi:10.3390/ijms22179603)
Supplement: Supplementary file 1 [file ijms-22-09603-s001.zip › Table S1.pdf]

Table S1: fold changes (Fc) of genes modulation vitamin D mediated depending on the presence of FokI receptor variants.

	**Basal**				**Inflamed**			
	**Up-regulated**		**Down-regulated**		**Up-regulated**		**Down-regulated**	
	FF	***Ff***	***FF***	***Ff***	***FF***	***Ff***	***FF***	***Ff***
A2M	3.41							0.43
ABCG1	4.62			0.41			0.49	
ACAN	2.63							
ACVR1C	2.27			0.37				0.40
ADAMTS15	26.74	2.17			2.44			0.08
ADAMTS4			0.47					0.39
ADAMTS9		2.46	0.39				0.46	
ADH1B	34.26				9.51			0.33
ADIG				0.36			0.21	
ADIPOQ			0.38	0.34				
AGTR1	2.96							0.38
ALCAM			0.42			2.04		
ALPL	2.41	2.62					0.41	
AMIGO2			0.08			14.33		
Amphiregulin				0.35				
ANGPTL4		2.09						0.48
ANKH	2.55							0.42
ANKRD1			0.20	0.50		3.76		
ANLN			0.24	0.49		10.39		
ANPEP			0.33			3.68		
ANXA6							0.33	
ANXA11	2.04							
APOE	4.95							0.35
APOL6	2.88							0.45
AQP1	7.26				3.61			0.34
ARHGAP26	4.64				2.24			0.34

ARPP21			0.30	0.34				0.35
ASPN	7.87						0.39	0.18
ATP1A2	6.59				3.45			0.13
ATRNL1	4.47	2.21						
BACE2			0.41			2.11		
BCAM	2.27							0.50
BCL2A1		2.40	0.28			3.28		
BCL2L13	2.61				3.30			0.45
BDNF			0.18			3.32		
BGLAP		2.17						0.34
BGN	2.35							0.34
BID			0.41			2.02		
BMP2		3.13	0.31			2.28		
BMP7	5.01			0.36				
BMPR1B			0.30				0.43	
BORA			0.37			2.63		
BST2		4.37	0.16		7.44	14.37		
C1QTNF1			0.18			4.83		
C2CD4A		2.42	0.21			2.52		
C7				0.34				
C7orf69			0.45	0.34	6.88	2.46		
CANX			0.41					
CCL13	3.48			0.33		2.83	0.49	
CCL2		3.14	0.10			2.97		
CCL20		21.60	0.01			29.30		
CCL3		2.97	0.09			6.01		
CCL4		4.37	0.22		2.07	3.38		
CCL5		5.73	0.01			24.11		
CCL7		2.41	0.11			2.87		
CCNA2			0.20			10.62		
CCR1	5.82	2.26						0.14

CCR2	2.01			0.34	2.71			0.08
CCR3	4.36	4.59					0.40	0.32
CCR5	3.75	2.10			57.89			
CCR6				0.34				
CD14	3.42	7.39						0.08
CD146		2.32	0.22			3.98		
CD168			0.18	0.49		9.61		
CD24	2.45							
CD271		2.47					0.39	
CD45				0.38	2.07			
CD54		2.22	0.39			2.30		
CD69				0.35				
CD73			0.46			2.01		
CDC42EP4	2.10							
CDH13			0.49					
CDH19			0.28	0.34		2.18		
CDH2				0.48				
CDKN1A	2.49							
CDS1	3.27	3.06						0.43
CEBPA	2.30	3.21						0.20
CES1	13.09	2.20						0.08
CFLAR		2.26						
CFTR				0.36				
CHI3L2								0.46
CLDN11	6.13			0.35			0.26	0.30
CLEC1A	2.05			0.35				
CLEC2B	2.70			0.09				0.44
CLEC3A				0.36	2.51			
CLEC3B	4.24			0.38				
CLGN						2.05		
CLIC6		2.41						

CLIP2	2.80			0.36				0.37
CMPK2		2.27	0.45		2.11	2.46		
CNR1				0.37				0.43
COL10A1	2.65	2.85						
COL11A1						2.07		
COL11A2	3.92	4.47					0.48	0.37
COL14A1	3.51							0.35
COL15A1	2.99			0.47	2.09			
COL2A1	3.79			0.39			0.38	0.39
COL4A1						2.91		
COL5A3	4.99				2.23			
COL9A1				0.34	2.23			
COL9A2	3.90							0.36
COL9A3	7.77			0.37				0.17
COMP	2.52	2.18					0.49	
CRHR1	4.07							
CRTAP	2.26	5.03						
CRYAB	2.21							
CSAD	2.15							
CSF1		2.18	0.16			2.62		
CSF2			0.00			13.60		
CSF3		7.53	0.05					
CSPG5					2.73			
CXCL1		20.02	0.00			7.13		
CXCL2		5.63	0.05			5.41		
CXCL5		3.94	0.03			2.24		
CXCL6		411.11	0.00			3.46		
CXCL8		78.95	0.00			7.24		
CXCR1		2.13	0.29			2.14		
CXCR2	3.50			0.33		3.29		
DCBLD1			0.43					

DCN	4.66			0.32				
DDR1								0.42
DGAT2	2.05						0.43	
DKK1			0.43					0.45
DNER			0.07			13.49		
DSG2	2.09							
DUOX1	4.38	4.27						0.23
EFEMP1		2.00	0.46					
EFNB2						3.02		
EHF			0.20	0.35		2.39	0.42	
ELN		9.63	0.42			2.28		
EPHX1	2.31							
EPYC	2.91			0.34			0.43	
F2RL1			0.38			3.47		
FABP4	2.57			0.34			0.26	0.23
FAT3								0.40
FBLN5	2.25							0.41
FGF18	3.19			0.48			0.41	0.25
FGF4				0.34				
FGF6				0.34				
FGF7		2.06	0.43					
FLRT2		3.87						
FMOD								0.37
FRZB	5.34			0.38	2.33			0.27
FZD3		2.34						0.39
FZD5		2.31						
FZD6			0.50					
FZD8			0.42					
GAB3	2.52	2.97						0.39
GABRB1			0.48			4.21		
GALNT15								0.41

GDF6			0.21			2.98		
GHR		2.27					0.45	0.46
GIMAP8	2.54			0.39		3.57		
GPC3	3.36			0.34				0.24
GPD1	2.09							0.39
GPD1L	2.24							0.48
GPR133	6.53	3.79						0.10
GPRC5B	6.68	2.21					0.41	0.18
GPT2			0.48					
HAPLN1			0.43			3.32		
HBB	3.14			0.37				0.37
HIF1A			0.48					
HLX	2.08							
HS3ST2	3.03				2.03			
HS6ST3	2.15	2.95						0.31
HSF2								0.47
HTR7	3.08							0.49
IBSP		9.52	0.04			60.87		
ICAM				0.34				
ID4	2.77							0.31
IDO1			0.17		2.52			0.50
IDO2	7.77			0.34				
IFI27		2.88	0.15		3.48	12.43		
IFI33L			0.10		4.08	3.86		
IFNG	2.09			0.34				
IGF1		4.41	0.31				0.47	
IGF1R								0.49
IGF2			0.41			3.57		
IGFBP1			0.27			7.13		
IGFBP2	2.05							0.42
IGFBP3			0.33					

IGFBP5	2.68							0.45
IHH	3.77			0.34	3.93			0.28
IL10	3.76			0.34	3.62	2.59		
IL11		6.20	0.09			11.97		
IL13				0.34			0.47	0.30
IL15			0.45					
IL15				0.34				
IL17A	2.33			0.34	3.01	4.22		
IL1A	2.42						0.36	0.22
IL1B		3.03	0.30					0.23
IL1R2	4.01			0.36	2.72	2.06		
IL1RN		3.21	0.22		2.71	2.14		
IL2	2.52			0.34				0.28
IL31RA	2.10			0.34				
IL32		2.30	0.15			2.88	0.27	
IL4				0.34				
IL5	6.42			0.34	2.03			
IL7	3.83				2.55			
INHA		2.10	0.37				0.41	0.45
ITGB8		2.58	0.23					
JAG1	3.59							
JPH3	2.95	2.13						0.42
KAT6A		2.63						
KCNE3				0.34			0.31	
KCNMB4				0.37				
KCNS3	2.28							
KRT14				0.44			0.21	
KRT18	2.06							
KRT19	4.91				3.37			
KYNU		4.12	0.08			8.96		
LAMP5						2.88		

LCN2		7.71	0.09			9.38		
LDB2	9.85						0.31	0.11
LDHA			0.41			2.24		
LEPR			0.34	0.35				0.35
LIF		2.02				2.82		
LMNB1			0.23	0.44		7.57		
LPAR4		5.77	0.39			2.99		
LPL	4.52							0.15
LRRC15			0.47		2.15	3.10		
LRRN3		5.02	0.19					
LRRN4CL	2.13							0.47
LSP1	3.17			0.39	3.49	2.99		
LYVE1	2.55			0.34		2.18		
MAB21L2	2.05			0.34				
MAP1LC3C	15.28				4.34			0.38
MAPK1	3.76							
MASP2	5.92			0.30	2.38			0.43
MATN2	2.08							0.42
MCAM		2.32	0.19			3.98		
MDM2	4.30							0.21
MET			0.48			2.71		
MFAP3L		3.20	0.27					
MFAP4	2.66							
MFAP5	2.97							0.32
MGP	2.78							0.29
MKI67			0.24	0.47	2.29	7.83		
MMP1		8.01	0.03			6.47		
MMP10		2.85	0.06			14.86		
MMP12		2.74	0.03			17.56		
MMP13		4.44	0.16			5.75		
MMP3		9.57	0.02			8.05		

MMP7		2.45	0.30					
MMP9			0.44					
MRAP	2.63			0.34	2.50			0.31
MXRA7	2.01							
MYBPH	2.93			0.41	4.89			0.43
MYL1	2.43			0.34				0.46
MYLIP	5.69							0.38
NAMPT		2.47	0.13			2.72		
NEFM			0.25			2.49	0.32	
NETO2			0.38			3.97		
NEURL3		2.32	0.22			3.64		
NFASC	2.50							0.46
NFKB1			0.45					
NLGN4Y							0.27	
NOD2		2.30	0.19			9.03		
NODAL	3.61			0.34		2.85	0.37	
NOS2		5.47	0.09			13.51		
NPTX2		2.55						
NR3C2	2.99							0.27
NR4A2	2.06							0.46
NR4A3		4.16						
NRM						2.14		
NRXN3				0.35				0.24
NTF4	4.06			0.33				
OGN	5.60							0.22
OLFML2A	2.90							0.42
OLFML2B	7.50							
OMD	6.96			0.32		4.17	0.34	
PBK			0.26	0.45		11.45		
PDE5A	2.75							0.35
PDGFRA	2.00							0.45

PDGFRB	4.14							0.32
PDIA4			0.44			2.04		
PENK			0.25			2.45	0.46	
PERP	3.62							0.17
PGF	3.56							0.24
PLCB4	2.91							
PLCB4		1.99						
PLIN1								0.47
PLPP3	2.04							
PODXL			0.17			9.51		
POSTN						2.90		
PPL	2.41							0.43
PRELP		3.93						0.24
PRL		15.20	0.01			12.98	0.40	
PTGDS			0.44					
PTGFR		3.65	0.25					
PTGS2		2.28						
PTHLH		3.53						
PTN	3.23							0.36
PTX3			0.18			3.15		
REEP1	4.07			0.41			0.32	0.29
RETN			0.37	0.35		2.17	0.39	
RNF128		2.05						0.50
RNF130			0.29	0.35				0.44
RSPO3		3.16	0.24					
S100A8				0.34		3.18		
S100A9				0.34		3.26		
SAA2		13.34	0.01			68.21		
SCARA5		6.01				2.40	0.30	
SCCA2				0.41	3.16			
SCD						2.00		

SDC4						2.13		
SCIN	4.99							0.21
SCRG1	5.46							
SEL1L	2.29	4.49						0.31
SEMA3F	3.16				2.03	2.24		
SEPP1	3.77	3.19						0.26
SERPINB2	3.30			0.37				0.11
SFRP1			0.23			3.07		
SGCG	2.59							0.25
SHISA2		2.61	0.23					
SIX1		2.07						
SLC16A3			0.33			2.23		
SLC24A1	5.41	2.90				2.10		
SLC27A2			0.37	0.36		2.15		
SLC40A1	19.42							0.09
SLC44A1	2.46						0.37	0.48
SLC7A1			0.44			2.45		
SLC7A2		7.61	0.04			16.00		
SLC7A7		2.02					0.49	
SLPI		2.63	0.40					
SMOC2	11.27							0.13
SNAI2	3.35							
SNED1			0.34					
SOSTDC1	2.42			0.34	2.11			
SOX4		2.08						
SOX9	2.61							
SPARC	2.54				2.34			
SRD5A3						2.11		
STC1		4.68	0.04			5.22		
STEAP4			0.22			4.07	0.38	
SYT4	3.39			0.36				0.26

TBX2						2.22		
TBX3			0.48					
TDO2				0.41	4.22			0.47
TENM1	2.12			0.35				
TERT				0.35	2.30			
TFAP2A				0.34		2.09		
TFR2		2.14	0.33			2.66	0.47	
TGFB1	2.82			0.36	2.33			0.31
TGFB3								0.37
THBS1			0.34			2.13		
THBS2	2.14							0.43
THBS3	2.07							
TIMP3	4.07							0.25
TLR2			0.10	0.35		2.07		
TMEM106A						2.56		
TMEM27	2.32	2.44					0.31	0.30
TMEM71		2.07	0.32			3.11		
TNFa		2.48	0.27			2.25	0.40	
TNFAIP6		10.83	0.03			5.03		
TNFAIP8		2.05	0.33			2.19		
TNFRSF11B			0.40			2.08		
TNFRSF1B			0.22			3.84		
TNMD	2.11			0.37				
TOP2A			0.22			9.49		
TP63			0.19	0.30	2.50	11.37		
TPH1	2.46			0.39				0.27
TSKU			0.35					
TUBB1				0.32				0.38
TUSC3	2.00							
TWIST2	2.51	2.24						0.41
UBE2B	2.18							0.47

VCAM1		2.09	0.21			2.30		
VEGFD	2.17	2.98			2.59			0.22
VEGFR				0.35				
VIT	7.02							0.26
WNT11		2.20	0.40			2.31	0.39	
