# Supplementary material for: High-Throughput Gene and Protein Analysis Revealed the Response of Disc Cells to Vitamin D, Depending on the VDR FokI Variants
Source: Int J Mol Sci. 2021 Sep 4;22(17):9603. doi: 10.3390/ijms22179603 (PMC8431769; doi:10.3390/ijms22179603)
Supplement: Supplementary file 1 [file ijms-22-09603-s001.zip › Table S2.pdf]

Table S2: detection range and sensitivity of analytes of the custom Human Magnetic Luminex Screening Assay..

Anlayte	Detection range (pg/mL)	Sensitivity (pg/mL)
BMP-2	16.5-4000	3.6
BMP-7	103-25000	8.56
IL-1Ra	28.8-7000	18.0
IL-2	30.9-7500	1.8
IL-3	78.2-19000	11.6
IL-6	4.53-1100	1.7
IL-17A	13.2-3200	1.8
MMP-1	49.4-12000	2.7
MMP-3	82.3-20000	5.3
MMP-13	144-35000	19.0
